# *Vibrio vulnificus*: An Environmental and Clinical Burden

**DOI:** 10.3389/fmicb.2017.00997

**Published:** 2017-05-31

**Authors:** Sing-Peng Heng, Vengadesh Letchumanan, Chuan-Yan Deng, Nurul-Syakima Ab Mutalib, Tahir M. Khan, Lay-Hong Chuah, Kok-Gan Chan, Bey-Hing Goh, Priyia Pusparajah, Learn-Han Lee

**Affiliations:** ^1^Jeffrey Cheah School of Medicine and Health Sciences, Monash University MalaysiaBandar Sunway, Malaysia; ^2^Division of Genetics and Molecular Biology, Faculty of Science, Institute of Biological Sciences, University of MalayaKuala Lumpur, Malaysia; ^3^Novel Bacteria and Drug Discovery Research Group, School of Pharmacy, Monash University MalaysiaBandar Sunway, Malaysia; ^4^Zhanjiang Evergreen South Ocean Science and Technology CorporationGuangdong, China; ^5^UKM Medical Centre, UKM Medical Molecular Biology Institute, Universiti Kebangsaan MalaysiaKuala Lumpur, Malaysia; ^6^Department of Pharmacy, Absyn University PeshawarPeshawar, Pakistan; ^7^Center of Health Outcomes Research and Therapeutic Safety (Cohorts), School of Pharmaceutical Sciences, University of PhayaoPhayao, Thailand

**Keywords:** *Vibrio vulnificus*, prevalence, pathogenesis, treatment, prevention

## Abstract

*Vibrio vulnificus* is a Gram negative, rod shaped bacterium that belongs to the family *Vibrionaceae*. It is a deadly, opportunistic human pathogen which is responsible for the majority of seafood-associated deaths worldwide. *V. vulnificus* infection can be fatal as it may cause severe wound infections potentially requiring amputation or lead to sepsis in susceptible individuals. Treatment is increasingly challenging as *V. vulnificus* has begun to develop resistance against certain antibiotics due to their indiscriminate use. This article aims to provide insight into the antibiotic resistance of *V. vulnificus* in different parts of the world as well as an overall review of its clinical manifestations, treatment, and prevention. Understanding the organism's antibiotic resistance profile is vital in order to select appropriate treatment and initiate appropriate prevention measures to treat and control *V. vulnificus* infections, which should eventually help lower the mortality rate associated with this pathogen worldwide.

## Introduction

The emergence of multidrug resistant strains of bacteria has become an international health crisis as illustrated by statements issued by the World Health Organization (WHO) highlighting antimicrobial resistance as a significant threat to human well-being (WHO, 2014). This crisis is largely attributable to indiscriminate use of antibiotics in clinical medicine as well in the agriculture and aquaculture industries. Antibiotic resistance is not something new, however the number of resistant pathogens, the geographic locations affected, and the extent of resistance in any particular organism are rising. All bacterial infections that were once believed to be treatable with appropriate antibiotics are returning in new leagues resistant to antibiotic therapy (Levy and Marshall, 2004).

There is no doubt that bacterial infection is a significant threat to mankind—human illness as a result of bacterial infection is common. *Vibrio* species including *Vibrio cholerae, Vibrio parahemolyticus*, and *Vibrio vulnificus* are among the common causes of foodborne infections in humans as a result of consumption of contaminated food, particularly seafood. Aside from this, these infections of aquatic livestock by organisms have also been responsible for large-scale losses in the aquaculture industry leading to prophylactic as well as therapeutic use of antimicrobials (Devi et al., 2009; Manjusha and Sarita, 2011; Letchumanan et al., 2014, 2015a). This excessive usage of antibiotics has caused the development of multidrug resistance in *Vibrio* species (Sudha et al., 2014; Letchumanan et al., 2015b).

While the emergence of multidrug resistant strains is already an alarming situation; this scenario is compounded by the dearth of new antibiotics in the pipeline (Rice, 2008; Freire-Moran et al., 2011). This article aims to provide insight into the antibiotic resistance of *V. vulnificus* in different parts of the world as well as an overall review of its clinical manifestations, treatment, and prevention. This information is vital in order to ensure that appropriate treatment is initiated and prevention measures are taken to treat and control *V. vulnificus* infections, with the aim of eventually lowering the mortality rate associated with this pathogen worldwide.

## Characteristic of *Vibrio vulnificus*

*V. vulnificus* belongs to the family *Vibrionaceae* and is found in warm coastal environments where water temperatures range from 9° to 31°C. The preferred habitat of *V. Vulnificus* has been reported to be water temperature in excess of 18°C with salinities between 15 and 25 parts per thousand (ppt) (Motes et al., 1998; Jones and Oliver, 2009; Huehn et al., 2014). Therefore, most cases of *V. vulnificus* infection are usually found in tropical or subtropical regions (Strom and Paranjype, 2000). *V. vulnificus* is classified into three biotypes based on their biochemical characteristics. Biotype 1 strains are predominantly responsible for severe human infection and are found worldwide in salt or brackish water. These biotype strains have been reported to be responsible for the entire disease spectrum mentioned earlier, including primary sepsis associated with the often-quoted fatality rate in excess of 50% (Horseman and Surani, 2011). Biotype 2 strains are primarily eel pathogens, found in saltwater in Eastern and Western Europe. Biotype 2 strains are capable of causing human infections but cases are rare (Strom and Paranjype, 2000; Oliver, 2005). Comparison of the genomic similarities among the three biotypes revealed biotype 3 to be a hybrid of biotypes 1 and 2. Biotype 3 strains are found in freshwater fish and their geographical range is limited to Israel (Jones and Oliver, 2009). Although biotype 3 is responsible for human infections and may cause serious infections requiring amputation, the reported mortality rate is <8% (Zaidenstein et al., 2008).

To cause human infection, *V. vulnificus* must first survive the inhospitable environment in our bodies and overcome our immune response. It is able to accomplish this due to its innate virulence factors/determinants which enhance its pathogenicity, conferring the ability to survive in the human body (Jones and Oliver, 2009). Severe mechanisms contribute in attachment and invasion of a host, which including pili, *OmpU*, and *IlpA* membrane proteins and flagella (Jung et al., 2005; Goo et al., 2006, 2007; Jones and Oliver, 2009). *V. vulnificus* are also able to utilize Neu5Ac as a nutrient during infection in humans and survive in highly acidic environments by breaking down amino acids to amines and CO_2_ (Jeong et al., 2009; Jones and Oliver, 2009). Surface expression of CPS is another virulence factor for *V. vulnificus* that enables it to survive in face of our immune response (Kashimoto et al., 2005). Besides that, RtxA1, Hemolysin (VvhA), and metalloprotease like VvpE and VvpM also contribute in cellular damage and cytotoxicity by causing haemolysis, cell apoptosis, and tissue necrosis, which result in bullous cutaneous lesions characteristics of systemic disease (Miyoshi and Shinoda, 2000; Lee et al., 2011; Lee M. A. et al., 2014).

## Occurrence of *Vibrio vulnificus*

*V. vulnificus* infections have been reported in diverse climate zones throughout the world (Table 1) including Denmark, Sweden, Germany, Spain, Turkey, Holland, Belgium, Israel, Italy, Korea, Japan, Taiwan, India, Thailand, Australia, and Brazil (Oliver et al., 1983; Dalsgaard et al., 1996; Bisharat et al., 1999; Torres et al., 2002; Oliver, 2006a,b, 2013; Patridge et al., 2009; Huehn et al., 2014; Karunasagar, 2014). This bacterium is commonly found in seafood samples with studies having reported that 3.5–8% of seafood samples in Europe, 2.4% of shrimp from Southeast Asia, 75% of freshly harvested oysters in India and 100% of oysters harvested from the Gulf of Mexico during warm months (May to October) contained *V. vulnificus* (Jones, 2014). Further, analysis of 180 cases in FDA records between 2002 and 2007 have shown that raw oysters is the main source of infection in the US with 92.8% of infected patients having consumed raw oysters. Studies have shown that there are 95 cases reported with 85 hospitalizations and 35 deaths per year globally (CDC, 2013).

**Table 1:** Occurrence of *Vibrio vunificus* cases, deaths, and mortality.

**Country**	**Source**	**Year**	**Reported cases**	**Deaths**	**Mortality rate (%)**	**Primary septicaemia (%)**	**Wound infections (%)**	**Association**	**References**
U.S.	Florida	1981–1992	125	44	67	57	28	Liver disease and immunocompromised	CDC, 1993
						fatality: 90.9			
	Florida	1981–1993	141	50	56	53	33	Liver disease, alcoholism	Hlady and Klontz, 1996
						fatality: 44	fatality: 5		
	23 states	1988–1996	422	143	38.4	43	45	Liver disease, alcoholism	Shapiro et al., 1998
						fatality: 61	fatality: 17		
	CDC	1997–2006	428	62	17	–	66	Liver disease	Dechet et al., 2008
	California	1991–2010	88	39	44.3	–	–	–	Vugia et al., 2013
France	New Caledonia	2008	3	3	–	–	–	Climatological events	Baker-Austin et al., 2016
Denmark	Danish Hospital	1994	11	1	9.1%	36.4	45.5	–	Dalsgaard et al., 1996
Israel	MOH	1995–1996	12	0	0	–	–	–	Bisharat and Raz, 1996
	–	1996–1997	62	0	0	–	100	Immunocompromised	Bisharat et al., 1999
Germany	Mecklenburg-Vorpommern	2003	2	1	–	–	100	Underlying chronic illnesses	Frank et al., 2006
	Mecklenburg-Vorpommern	2006	3	0	0	–	100	Underlying chronic illnesses	Koch, 2004
Greece		1997–2003	9	2	–	–	100	–	Mouzopoulos et al., 2008
Japan	–	1978–1987	38	26	68	60.5	7.9	Liver disease, leukopenia	
	–	1999–2003	94	58	61.7	72.3	22.3	Alcoholic liver disease	Inoue et al., 2008
					fatality: 75				
	Coastal of Ariake Sea	1984–2008	37	24	64.9	–	0	Liver disease	Matsumoto et al., 2010
					fatality: >60%				
	Saga Hospital	2001–2010	12	7	58.3	–	–	Underlying diseases	Matsuoka et al., 2013
Korea	MOH	2001–2010	588	285	48.5	–	–	Liver disease, alcoholism	Lee et al., 2013
	Chosun Hospital	2000–2011	34	16	–	63	–	–	Yun et al., 2015
Taiwan	–	1985–1990	27	11	42.3	64.3	28.6	–	Chuang et al., 1992
						fatality: 5.6	fatality: 12.5		
	Chang Gung Hospital	NA–1994	18	10	55.6	77.8	22.2	Liver disease	Chang et al., 1996
	–	1995–2003	93	31	33.0	5.4	36.6	–	Liu et al., 2006
	Chang Gung Hospital	2002–2007	23	7	30.0	–	100	–	Tsai et al., 2009
	–	2007–2010	36	4	11.1	–	100	–	Tsai et al., 2012
	Chi Mei Hospital	1998–2011	121	35	29.0	–	–	–	Chao et al., 2013
	National Medical Center	1996–2011	140	18	18.0	58	78	–	Lee M. A. et al., 2014

In the United States, *V. vulnificus* is a leading cause of seafood-associated fatality. There are ~50 cases of *V. vulnificus* infection with 45 hospitalizations and 16 deaths every year (CDC, 2013). *V. vulnificus* is found to be isolated from Atlantic and Pacific coasts where 74% of retail oysters in the U.S. contained *V. vulnificus* with the greatest detection frequency from the Gulf of Mexico (Texas, Louisiana, Mississippi, Alabama, and Florida), followed by Mid-Atlantic, North Atlantic, and the Pacific (Oliver, 2006a,b; Jones, 2014). In addition, *V. vulnificus* can replicate itself in postharvest seafood if it is not cooled immediately, therefore the *V. vulnificus* level is greater at the time of consumption (retail, market) rather than at harvest (Jones, 2014). *V. vulnificus* found in the Gulf Coast region with a peak in the onset of both systemic and wound infection between April to September because *V. vulnificus* favors tropical and subtropical region (Strom and Paranjype, 2000). The Florida Department of Health and Rehabilitative Services (HRS) reported 125 confirmed cases of *V. vulnificus* from 1981 to 1992 (Hlady and Klontz, 1996). As shown in Table 1, there were 422 cases from 23 states reported to the Centers for Disease Control and Prevention (CDC) between 1988 and 1996; out of these, 45% were wound infections, 43% were primary septicaemia, and the associated mortality was 38.4% (Amaro and Biosca, 1996; Hlady and Klontz, 1996). It is likely that the actual incidence of cases of *V. vulnificus* gastroenteritis in the community is higher than documented but that the majority of cases go unreported since the illness is seldom severe enough to require hospitalization (Strom and Paranjype, 2000). Vugia et al. (2013) reported 88 cases of *V. vulnificus* infection in California with a total of 39 deaths from 1991 to 2010.

In New Caledonia, three deaths due to *V. vulnificus* infection were reported in 2008, all suspected to have been acquired via ingestion of contaminated oysters. All three patients died from primary septicaemia and was significant enough to attract attention as *V. vulnificus* infections are rare in the South Pacific (CDC, 2011). It was hypothesized that the salinity in Caledonia was significantly reduced due to the heavy precipitation in early 2008, which then indirectly provided a fertile environment for the organisms to proliferate. These examples further suggest that changes in climate may be a contributory factor in increasing risk of *V. vulnificus* infection (Baker-Austin et al., 2016).

From August 1995 to summer 1996, 25 cases of *Vibrio* infections were reported to the Epidemiology Department at the Ministry of Health in Israel; out of which 12 cases identified to be *V. vulnificus* (Bisharat and Raz, 1996). There was a subsequent upsurge with 62 cases reported between May 1996 and December 1997. Of these 62 cases, 33 were confirmed cases and 29 were suspected cases without laboratory confirmation; 57 (92%) developed cellulitis, four with necrotising fasciitis, and one with osteomyelitis, but fortunately, no deaths were reported. Nevertheless, both wound infection and bacteraemia were identified for all 62 cases (Bisharat et al., 1999).

In summer 2003, two cases of *V. vulnificus* infection were reported in Mecklenburg-Vorpommern, Germany. Both patients had open wounds on the legs and one patient died after developing the infection (Frank et al., 2006). In the same region in Germany, three cases of *V. vulnificus* infection were reported in summer 2006, all involving patients with wound infections who were treated with antibiotics (Koch, 2004). A retrospective study looking at the time period between June 1997 and July 2003, identified nine patients with lower extremity *V. vulnificus* infection, all of whom had associated foot injuries. Postoperative complications led to two fatalities while others were all treated with antibiotics or amputation (Mouzopoulos et al., 2008).

Unsurprisingly, there is geographical variation in the primary source of infection based on dietary differences. For example, a mud shrimp, *Upogebia major* rather than raw oysters represent the primary source of *V. vulnificus* infection in Japan (Karunasagar, 2014). It is possible that other kinds of seafood may also cause infection as demonstrated by a study conducted in a Chinese local seafood market which found that 100% of razor clams, 100% of tiger prawns, and 56% of shrimp, were contaminated with *V. vulnificus* with bacterial levels up to ~70,000 per gram (Jones, 2014). In Japan, a questionnaire—based retrospective study was conducted in 1,693 hospitals, which showed a total of 93 reported cases of *V. vulnificus* infection in Japan between 1999 and 2003. Out of this total, 68 cases (72.3%) presented with septicaemia and the mortality rate was 75%. The prognosis of primary septicaemia was found to be worse than that of wound infection (*P* < 0.001; Inoue et al., 2008). Recently, Matsuoka et al. (2013) investigated and reported 12 patients with *V. vulnificus*-induced necrotic fasciitis—7 out of 12 died, giving a mortality rate of 58.3% and without exception, all patients involved had underlying diseases (Matsuoka et al., 2013).

In neighboring Korea, the National Notifiable Diseases Surveillance System reported 588 confirmed *V. vulnificus* infection cases from 2001 to 2010. Out of all cases reported, 285 were fatal, giving a mortality rate of 48.5% (Lee et al., 2013). In a separate report, 34 cases were reported in Chosun University Hospital from January 2000 to December 2011, with 16 fatalities (Yun et al., 2015). In Chang gung Memorial Hospital, Taiwan, 18 patients were identified with *V. vulnificus* infection over an unspecified period of time. Of all cases, 14 patients manifested with primary septicaemia and four patients with wound infection with an overall mortality of 55.6% in this study (Chang et al., 1996). As seen in Table 1, from January 2007 to June 2010, among 143 patients with necrotizing fasciitis, 36 patients were identified to have *V. vulnificus* infections. Of all the patients with *V. vulnificus* infections, there were four fatalities yielding a mortality rate of 11.1% (Tsai et al., 2012). In Chi Mei Medical Center, 121 patients who were confirmed with *V. vulnificus* between July 1998 and June 2011 were recruited into a study. Of all patients with infections, 35 patients died yielding a mortality rate of 29% (Chao et al., 2013).

## Healthcare burden

Bacterial infections are a constant strain/burden on the healthcare sector. In the U.S., the annual health costs of seafood-related diseases were calculated to be US$ 350 million with direct exposure to *V. vulnificus, Vibrio parahaemolyticus, Vibrio alginolyticus*, and aerosolized *Karenia brevis* accounting for over US$30 million. Studies have shown that premature deaths (i.e., death before the age of 75 years) accounted for a large proportion of the total cost of seafood-related illness treatment costs (US$306 million), followed by medical care (US$25 million), hospitalization (US$6 million), and loss of productivity (US$15 million), and these inevitably become a great healthcare burden to the U.S. (Ralston et al., 2011).

On further breakdown, *V. vulnificus* had the dubious honor of being the costliest marine-borne pathogen in the study with an annual cost of illness 10 times higher than any other pathogen, accounting for 66% of seafood-related illness health costs and 26% of the total health costs. Even more worryingly, *V. vulnificus* accounts for 1/3 of the total seafood-related illness and more than 85% of the costs of direct exposure to *Vibrio* pathogen. These were due primarily to a high rate of premature death with a mortality rate of 31% for seafood-related infection and 18% for infections from direct exposure. This should garner the significant attention as premature death costs US$ 238 million, which accounts for 99% of the total *V. vulnificus* health costs and 75% of the total cost of premature death (Ralston et al., 2011).

*V. vulnificus* are usually susceptible to most antibiotics of veterinary and human significance (Oliver, 2006a). However, the extensive use of antibiotics in health care, agriculture, and aquaculture systems has led to the increase in antibiotic resistance in many bacterial genera including *Vibrio* species over the past few decades (Cabello, 2006; Letchumanan et al., 2015a,b,c). *V. vulnificus* in seafood and aquatic environments are exhibiting resistance to multiple antibiotics due to the misuse of antibiotics (Elmahdi et al., 2016). As multiple-antibiotic resistant bacteria pose a threat in both to fish and shellfish farming and human health, appropriate actions need to be taken as it raises serious public health and economic concerns (WHO, 2014). *V. vulnificus* resistance toward common antibiotics has reached alarming levels in many countries (Table 2) which has serious implications on the treatment methods for bacterial infections (WHO, 2014).

**Table 2:** Geographical distribution of antibiotic resistance profiles of *Vibrio vulnificus*.

**Country**	**Source**	**Year**	**Isolates**	**Susceptible**		**Resistant**		**References**
United States	Louisiana Gulf and retail oysters	2005–2006	151	**Penicillins and B-lactamase inhibitor combination** Ampicillin **Tetracycline Cephems** Cefotaxime Ceftazidime **Quinolones** Fluoroquionolones		–		Shaw et al., 2014
	Chesapeake Bay and Maryland Coastal Bays	2009	120	**Penicillins and B-lactamase inhibitor combination** Ampicillin (115/120) Amoxicillin (118/120) Ampicilin-sulbactam (120/120) Penicillin (116/120) Piperacillin (119/120) **Carbapenems** Imipenem (118/120) Meropenem (120/120) **Aminoglycosides** Amikacin (117/120) Apramycin (106/120) Gentamicin (120/120) Streptomycin (90/120)	**Cephems** Cefepime (120/120) Cefotaxime (120/120) Cefoxitin (108/120) Ceftazidime (118/120) Ceftriaxone (119/120) Cefuroxime sodium (120/120) Cephalothin (114/120) **Tetracyclines** Doxycycline (120/120) Tetracycline (119/120) **Quinolones** Ciprofloxacin (120/120) Levofloxacin (120/120) Oflaxacin (120/120) **Others** Chloramphenicol (26/120) Trimethoprim-sulfamethoxazole (120/120)	I: **Penicillins and B-lactamase inhibitor combination** Ampicillin (3/120) Piperacillin (1/120) **Cephems** Cefoxitin (6/120) Ceftazidime (2/120) Cephalothin (4/120) **Aminoglycosides** Amikacin (3/120) Apramycin (9/120) Streptomycin (20/120) **Others** Chloramphenicol (94/120)	R: **Penicillins and B-lactamase inhibitor combination** Ampicillin (1/120) Penicillin (4/120) Piperacillin-tazobactam (1/120) **Cephems** Cefoxitin (5/120) Cephalothin (2/120) **Carbapenems** Imipenem (1/120) **Aminoglycosides** Apramycin (5/120) Streptomycin (8/120)	Shaw et al., 2014
	South Caroline and Georgia			Doxycycline, tetracycline, aminoglycosides and cephalosporin				Baker-Austin et al., 2009
Brazil	Coreaú Rive	2005–2006	1	**Carbapenem** Imipenem (1/1) **Cephems** Cefoxitin (1/1) Nitrofurantoin (1/1) **Monobactam** Aztreonam (1/1) **Tetracycline** Oxytetracycline (1/1) Tetracycline (1/1)	**Quinolones** Nalidixic acid (1/1) **Aminoglycosides** Gentamicin (1/1) **Others** Sulfamethoxazole (1/1) Florfenicol (1/1)	**Penicillin and B lactamase inhibitor** Ampicillin (1/1)		Reboucas et al., 2011
Italy	Coastal water of Northern Sardinia	NA	6	**Carbapenem** Imipenem (3/6) **Others** Trimethoprim-sulfamethoxazole (6/6)		**Penicillins and B-lactamase inhibitor combination** Ampicillin (3/6) **Tetracycline** Doxycycline (1/6)		Zanetti et al., 2001
	Coastal water, Adriatic Sea	NA	8	**Tetracycline** (8/8) Oxytetracycline (6/8) Doxycycline (8/8) **Carbapenem** Imepenem (8/8) Meropenem (8/8) **Polymyxin** Colistin (4/8) Polymyxin (7/8) **Quinolones** Flumequin (7/8) Oxolinic acid (8/8) Nalidixic acid (6/8) Ciprofloxacin (8/8)	**Aminoglycosides** Kanamycin (2/8) Neomycin (4/8) **Cephems** Cefotaxime (7/8) Nitrofurantoin (6/8) **Others** Choramphenicol (8/8) Lincomycin (1/8) Novobiocin (3/8) Trimethoprim-sulfamethoxazole (7/8) Sulfamethoxazole (3/8) Trimethoprim (8/8) Rifampicin (3/8)	I: **Penicillins and B-lactamase inhibitor combination** Penicillin (2/8) Carbenicillin (2/8) Ampicillin (2/8) **Aminoglycosides** Streptomycin (1/8) Kanmycin (2/8) Neomycin (2/8) **Quinolones** Nalidixic acid (2/8) Flumequin (1/8) **Cephems** Nitrofurantoin (1/8) Cefotaxime (1/8) Cephalothin (3/8) **Polymyxin** Colistin (2/8) **Others** Novobiocin (2/8) Trimethoprim-Sulfamethoxazole (1/8) Rifampicin (2/8)	R: **Penicillins and B-lactamase inhibitor combination** Penicillin (6/8) Carbenicillin (6/8) Ampicillin (6/8) **Aminoglycosides** Streptomycin (7/8) Kanamycin (4/8) Neomycin (2/8) **Cephems** Cephalotin (3/8) **Polymyxin** Colistin (2/8) Polymyxin (1/8) **Tetracycline** Oxytetracycline (2/8) **Others** Lincomycin (7/8) Novobiocin (3/8) Sulfamethoxazole (5/8) Rifampicin (3/8)	Ottaviani et al., 2001
Germany	Baltic Sea and North Sea coastline, estuaries of rivers Ems and Weser	2004–2014	141 E =122 (susceptible = 72, non-susceptible = 50) C = 19 (susceptible = 8, non-susceptible = 11)	**Quinolones** (141/141) Fluoroquinolone (141/141) **Tetracycline** (141/141) **Folate pathway inhibitor** (141/141)	**Others** Phenicols (141/141) **Carbapenems** (141/141) **Cephalosporin** (141/141) **Penicillins and B-lactamase inhibitor combination** Aminopenicillin with or without B-lactamase inhibitors (141/141)	I: **Aminoglycosides** Streptomycin (58/141) Kanamycin (1/141)	R: **Aminoglycosides** Streptomycin (4/141)	Bier et al., 2015
China	Retail market in Hangzhou	2012	33	**Penicillins and B-lactamase inhibitor combination** Ampicillin (33/33) Ampicillin-sulbactam (33/33) Piperacillin-tazobactam (33/33) **Carbapenems** Imipinem (33/33) **Others** Trimethoprim-sulfamethoxazole (33/33) Chloramphenicol (33/33)	**Cephems** Cephalothin (33/33) Cefotaxime (33/33) Ceftazidime (33/33) Ceftriaxone (33/33) Nitrofurantoin (33/33) **Quinolones** Nalidixic acid (33/33) Ciprofloxacin (33/33) Levofloxacin (33/33)	I: **Aminoglycosides** Streptomycin (15/33) Gentamicin (31/33) Tobramycin (33/33) **Cephems** Cefazolin (33/33)	R: **Aminoglycosides** Gentamicin (31/33) Streptomycin (15/33) Tobramycin (33/33) **Cephems** Cefazolin (33/33) Cefepime (1/33) **Monobactam** Aztreonam (8/33) **Tetracycline** (2/33)	Pan et al., 2013
Hong Kong	*Sparus sarba*	1995–1997	16 isolates 4 strains	**Cephems** Ceftriaxone (12/12) **Aminoglycosides** Streptomycin (12/12)	**Quinolones** Nalidixic acid (12/12) **Others** Rifampicin (12/12)	**Penicillins and B-lactamase inhibitor combination** Ampicillin (1/4) **Cephems** Cefuroxime (1/4)	**Aminoglycosides** Gentamicin (2/4) Amikacin (3/4) Kanamycin (4/4) Netilimicin (2/4) **Tetracycline** (1/4) **Others** Trimethoprim (1/4)	Li et al., 1999
India	Cochin	2010–2011	2	**Quinolones** Nalidixic acid (2/2) **Tetracycline** (2/2) **Others** Chloramphenicol (2/2)		**Penicillins and B-lactamase inhibitor combination** Ampicillin (2/2) Amoxycillin (2/2) Carbenicillin (2/2) **Polymyxin** Colistin (2/2)	**Cephems** Ceftazidime (2/2) Cephalothin (2/2) **Aminoglycosides** Streptomycin (2/2)	Sudha et al., 2014
	East Coast	1999–2002	7	**Penicillins and B-lactamase inhibitor combination** Penicillin G (1/7) **Tetracycline** Chlortetracycline (4/7) Oxytetracycline (1/7) **Quinolones** Ciprofloxacin (2/7)	**Aminoglycosides** Kanamycin (1/7) **Polymyxin** Polymyxin B (1/7) **Others** Chloramphenicol (3/7) Erythromycin (5/7)	I: **Tetracycline** Chlortetracycline (2/7) Tetracycline (5/7) Oxytetracycline (4/7) **Cephems** Ceftriaxone (2/7) **Quinolones** Ciprofloxacin (2/7) Nalidixic acid (4/7) **Penicillins and B-lactamase inhibitor combination** Penicillin G (1/7) **Aminoglycosides** Gentamicin (2/7) Streptomycin (3/7) Kanamycin (3/7) Neomycin B (4/7) **Polymyxin** Polymyxin B (4/7) **Others** Chloramphenicol (4/7) Erythromycin (2/7) Furazolidone (3/7)	R: **Tetracycline** Chlortetracycline (1/7) Tetracycline (2/7) Oxytetracycline (2/7) **Cephems** Ceftriaxone (5/7) **Quinolones** Ciprofloxacin (3/7) Nalidixic acid (3/7) **Penicillins and B-lactamase inhibitor combination** Ampicillin (7/7) Penicillin G (5/7) **Aminoglycosides** Gentamicin (5/7) Kanamycin (3/7) Neomycin B (4/7) Streptomycin (4/7) **Polymyxin** Polymyxin B (2/7) **Others** Furazolidone (4/7)	Vaseeharan et al., 2005
Philippine	Shrimp farms	NA	14	**Quinolones** Oxolinic acid (7/14) **Tetracycline** Oxytetracycline (7/14) **Others** Furazolidone (7/14) Chloramphenicol (7/14)		**Tetracycline** Oxytetracycline (3/7)		Tendencia and de la Peña, 2001
Korea	Fish markets and estuarine sites	2009	31 (seafood = 17, *E* = 14)	**Penicillins and B-lactamase inhibitor combination** Ampicillin (11/31) Amoxicillin (23/31) Ampicillin-sulbactam (26/31) Piperacillin (11/31) Piperacillin-tazobactam (17/31) **Carbapenem** Imipenem (28/31) Meropenem (22/31) **Quinolones** Ciprofloxacin (13/31) Levofloxacin (21/31) Ofloxacin (22/31) Enrofloxacin (19/31)	**Cephems** Cephazolin Ceftazidime (21/31) Cephazolin (10/31) Cefepime (19/31) Cefotaxime (15/31) Cefoxitin (12/31) Cefuroxime sodium (12/31) Cephalotin (10/31) **Aminoglycosides** Amikacin (13/31) Gentamicin (21/31) **Tetracycline** (19/31) **Others** Chloramphenicol (23/31) Trimethoprim-sulfamethoxazole (25/31)	I: **Penicillins and B-lactamase inhibitor combination** Ampicillin (2/31) Amoxicillin (7/31) Ampicillin-sulbactam (3/31) Piperacillin (9/31) Piperacillin-tazobactam (10/31) **Cephems** Cephazolin (3/31) Cefepime (7/31) Cefotaxime (10/31) (8/31) Ceftazidime (4/31) Cefuroxime sodium (8/31) Cephalotin (10/31) **Carbapenem** Imipenem (3/31) Meropenem (5/31) **Aminoglycosides** Amikacin (6/31) entamicin (4/31) **Tetracycline** (7/31) **Quinolones** Ciprofloxacin (9/31) Levofloxacin (6/31) Ofloxacin (6/31) Enrofloxacin (7/31) **Others** Chloramphenicol (5/31) Trimethoprim-sulfamethoxazole (2/31)	R: **Penicillins and B-lactamase inhibitor combination** Ampicillin (18/31) Amoxicillin (1/31) Ampicillin-sulbactam (2/31) Piperacillin (11/31) Piperacillin-tazobactam (4/31) **Cephems** Cephazolin (18/31) Cefepime (5/31) Cefotaxime (6/31) (11/31) Ceftazidime (6/31) Cefuroxime sodium (11/31) Cephalotin (11/31) **Carbapenem** Meropenem (4/31) **Aminoglycosides** Amikacin (12/31) Gentamicin (6/31) **Tetracycline** (5/31) **Quinolones** Ciprofloxacin (9/31) Levofloxacin (4/31) Ofloxacin (3/31) Enrofloxacin (5/31) **Others** Chloramphenicol (3/31) Trimethoprim-sulfamethoxazole (4/31)	Kim et al., 2011
South Africa	Wastewater treatment facility	NA	18	**Carbapenems** Imipenem (18/18) Meropenem (18/18) **Quinolones** Norfloxacin (18/18)		**Penicillins and B-lactamase inhibitor combination** Ampicillin (18/18) **Others** Sulfamethoxazole (18/18)	**Cephems** Cephalothin (17/18)	Okoh and Igbinosa, 2010

In the U.S., *V. vulnificus* is a leading cause of seafood-associated fatalities; there are ~50 cases of *V. vulnificus* infection with 45 hospitalizations and 16 deaths every year (CDC, 2013). Primary septicaemia, which is the main characteristic of *V. vulnificus* infection in humans is associated with a high mortality rate (Feldhusen, 2000). A study in South Carolina and Georgia involving clinical and environmental *V. vulnificus* isolates showed that 45% of the environmental isolates were resistant to three or more classes of antibiotics while 17.3% were resistant to 8 or more antibiotic agents. This includes the drugs that are usually prescribed for *V. vulnificus* infections, including doxycycline, tetracycline, aminoglycosides, and cephalosporin (Baker-Austin et al., 2009).

In Germany, *Vibrio* infections occur sporadically but incidence peaks after extreme heatwaves (Huehn et al., 2014). Bier et al. (2015) studied the antibiotic resistance profile of *V. vulnificus* and *V. cholerae* that were recovered along the Baltic Sea and North Sea coastline as well as the estuaries of the rivers Ems and Weser. Results showed that *V. vulnificus* isolates were susceptible to all the antimicrobial agents tested including quinolones, tetracycline, folate pathway inhibitors, carbapenems, cephalosporins, and aminopenicillins with or without B-lactamase inhibitors, while only 2% of the isolates showed resistance toward streptomycin (Bier et al., 2015). This results showed a variation in the antibiotic resistance pattern when compared with the findings from Baker-Austin et al. (2009). This suggest that the usage of antibiotics in different aquaculture and clinical settings influence the resistance pattern among the *Vibrio* species.

The antibiotic resistance profile of *V. vulnificus* has also been studied in Asian countries. In China, the antibiotic resistance profile was studied in 33 *V. vulnificus* isolates from retail shrimps in Hangzhou which were tested against 21 antibiotics. The findings showed that they were resistant or intermediately resistant to cefepime (3.03%), tetracycline (6.06%), aztreonam (24.24%), streptomycin (45.45%), gentamycin (93.94%), tobramycin (100%), and cefazolin (100%). Fortunately, all the isolates obtained were sensitive to ampicillin, ampicillin-sulbactam, piperacillin-tazobactam, cephalothin, ceftriaxone, cefetaxime, ceftazidime, imipenem, ciprofloxacin, levofloxacin, nalidixic acid, trimethoprim-sulfamethoxazole, chloramphenicol, and nitrofurantoin (Pan et al., 2013).

Li et al. (1999) reported the antibiotic susceptibility of 51 *Vibrio* strains collected from *Sparus sarba* from May 1995 to February 1997 in Hong Kong. The study found that all strains were sensitive to ceftriaxone, streptomycin, nalidixic acid, and rifampicin. However, there four strains that were resistant to ampicillin, cefuroxime, tetracycline, trimethoprim, and aminoglycosides including gentamicin, amikacin, kanamycin, netilimicin (Li et al., 1999).

In India, between September 2010 and March 2011, a study of pathogenic *Vibrio* strains which made up 2% of *V. vulnificus* isolates from four retail markets in Cochin, India were tested for their susceptibility to various classes of antibiotics. This study showed that all *V. vulnificus* isolates were susceptible to chloramphenicol, tetracycline, and nalidixic acid, and were all resistant to ampicillin, amoxicillin, carbenicillin, colistin, ceftazidin, cephalothin, and streptomycin (Sudha et al., 2014).

## Clinical manifestation

*V. vulnificus* can cause severe, potentially life-threatening infection in susceptible patients. This bacterium is transmitted via seafood handling or consumption of contaminated seafood, especially raw or undercooked oysters or through direct inoculation into open wounds (Gulig et al., 2005; Dechet et al., 2008; Jones and Oliver, 2009; Daniel, 2011). Gastroenteritis usually results from the consumption of contaminated seafood (raw oysters) and the patient may present with nausea, vomiting, and abdominal pain which often progresses to fever, chills, and cutaneous manifestations (Haq and Dayal, 2005). Since these cases generally do not result in systemic shock or localized cellulitis, the vast majority of these cases are unreported.

In certain cases, *V. vulnificus* infection can be fatal particularly when it results in primary septicaemia and necrotizing fasciitis (Strom and Paranjype, 2000). Septicaemia appears to be the most common or in some data a close second presentation of infection, and has the worst outcome with mortality rate of more than 50% (Hlady and Klontz, 1996; Feldhusen, 2000). The portal of entry is believed to be the small intestine or cecum, but the ileum is considered the most likely site (Chen et al., 2002). Primary septicaemia is characterized by bacteremia without any obvious focus of infection and usually presents with sudden onset of fever and chills, often accompanied by vomiting, diarrhea, abdominal pain, and pain in the extremities within 7 days after ingestion of contaminated seafood; though symptom onset might be delayed for up to 14 days (Chen et al., 2002; Haq and Dayal, 2005). Within the first 24 h after the onset of illness, secondary cutaneous lesions such as cellulitis, bullae, and ecchymoses begin to appear on patient's extremities (Haq and Dayal, 2005). Besides that, septic shock (systolic blood pressure <90 mmHg), mental status changes (obtundation, lethargy, or disorientation), and thrombocytopenia were also reported in patients with primary septicaemia (Blake et al., 1979; Klontz et al., 1988; Shapiro et al., 1998). Primary septicaemia is fatal in 60–75% of cases with development of hypotension within 12 h of admission representing a particularly poor prognostic factor, as these cases are twice more likely to die compared to infected patients with normal blood pressure (Klontz et al., 1988).

Necrotizing fasciitis usually results from handling contaminated seafood or exposure of open wounds to contaminated water (Klontz et al., 1988; Dechet et al., 2008; Jones and Oliver, 2009). Severity of the infection may vary from mild to severe with symptoms occurring within 7 days, but could also be delayed as long as 12 days from exposure (Horseman and Surani, 2011). Wound infection can advance to cellulitis and become necrotic but fortunately its fatality rate is lower than primary septicaemia, ranging from 20 to 30% (Strom and Paranjype, 2000; Karunasagar, 2014). In contrast to primary septicaemia, necrotizing fasciitis is limited to the affected area and metastatic infection is not observed (Horseman and Surani, 2011).

Besides the above-mentioned presentations, it should be noted that patients may also have atypical presentations including spontaneous bacterial peritonitis, pneumonia, endometritis, meningitis, septic arthritis, osteomyelitis, endophthalmitis, and keratitis, all of which have been reported in recent years (Penland et al., 2000; Johnson and Arnett, 2001; Jung et al., 2005).

## Treatment and prevention

Prompt treatment with appropriate antibiotics is essential for optimal patient outcomes, particularly in more severe manifestations such as systemic septicaemia and wound infections (Krovacek et al., 1994; Moreno and Landgraf, 1998). Regardless of the route of infection, *V. vulnificus* infection responds positively to antibiotics and it has been clearly demonstrated that the greater the delay in the initiation of treatment, the higher the fatality rate (Rodrigues et al., 1992; Amaro et al., 1994; Krovacek et al., 1994; Moreno and Landgraf, 1998). Several classes of antimicrobials are suitable for treatment of *V. vulnificus* infection. According to the CDC, doxycycline, and ceftazidime are the antibiotics of choice for *V. vulnificus* infections in adults, while in children with *V. vulnificus* wound infection, the recommended treatment is trimethoprim-sulfamethoxazole (co-trimoxazole) and an aminoglycoside while doxycycline and fluoroquinolones are contraindicated (CDC, 2013). Infectious Diseases Society of America (IDSA) suggests doxycycline with ceftriaxone or cefotaxime as the first-line antimicrobial agent in adults with *V. vulnificus* infections (Stevens et al., 2014).

However, the first-line antimicrobial agents suggested above may no longer be applicable to all countries with *V. vulnificus* infection. By correlating both the reported resistance profile mentioned in Table 2 and the recommended treatment by IDSA and CDC, antibiotics should be tailored in different countries as the first-line antimicrobial agent might no longer be applicable to patients with *V. Vulnificus* infection. For example, doxycycline, the antibiotic that is been suggested as first-line treatment by both the CDC and IDSA has shown intermediate resistant profile (1/6) in Italy (Zanetti et al., 2001). Where else, ceftazidime, one of the first-line treatment measures suggested by the IDSA has been reported to have an intermediate resistant profile (2/120) in the U.S. (Shaw et al., 2014) and resistant (2/2) in India (Sudha et al., 2014). Therefore, based on the current evidence, a more appropriate choice would be cefotaxime and ceftriaxone. However, even here there are challenges as ceftriaxone, one of the drugs of choice from the group cephem that has been chosen as one of the first-line drug, also shows an intermediate resistant (2/7) to a resistant profile (5/7) in India (Vaseeharan et al., 2005). As for children with *V. vulnificus* infection, co-trimoxazole has an intermediate resistant profile (1/8) in Italy and resistant profile (8/8) in South Africa. While aminoglycosides are also safe to use in children with *V. vulnificus* infection, it has an intermediate resistant and resistant profile in Italy, Germany, and China; and resistant profile alone in Hong Kong and India. With all the evidence, it is clearly vital for clinicians and microbiologists to work with policy makers to review updated resistance profile in that particular country in order to facilitate usage of the most appropriate antibiotics to maximize the treatment efficacy.

Other than conventional medication mentioned above, it is essential for patients with severe soft tissue infection to undergo surgery (Hsueh et al., 2004; Park et al., 2009) as antimicrobial therapy is usually ineffective due to the thrombosis of the blood vessels supplying the infected area (Horseman and Surani, 2011). Aggressive surgical debridement or amputation is necessary to remove necrotic tissues and the prognosis is good when early debridement and fasciotomy are performed (Kuo et al., 2007).

CDC has published recommendations to prevent *V. vulnificus* infections. Among the preventive measures to be taken are to avoid eating raw oysters or shellfish especially those harvested from warm salt and brackish water, avoid exposure of open wounds to warm salt and brackish water, wear protective clothing (gloves) when handling raw shellfish, and wear protective footwear (wading shoes) when wading in warm salt or brackish water (French et al., 1989).

*V. vulnificus* can rapidly multiply in harvested seafood if it is not cooled immediately. Without proper storage, *V. vulnificus* levels may be significantly increased when the seafood reaches point of sale and eventual consumption (retail, market) compared to at harvest (Jones, 2014). According to U.S. regulations, commercial shellfish should be refrigerated within 10 h of harvest when the water temperature exceeds 27°C because temperature seems to have a role in influencing bacterial concentration (Ghazaleh et al., 2014; Karunasagar, 2014). Freezing combined with frozen storage and high hydrostatic pressure are the recommended postharvest treatment for oysters in the U.S. (Karunasagar, 2014). Based on FAO/WHO risk assessment, the Codex Committee on Food Hygiene has also developed a Code of Hygienic Practice for control of *Vibrio* spp. in seafood with an annex on control measures for *V. vulnificus* in bivalve molluscs (Karunasagar, 2014). U.S. FDA and the Interstate Shellfish Sanitation Conference regulate the hazard analysis critical control points (HACCP) for time or temperature controls in order to minimize the exposure of shellfish to elevated temperatures and include defined minimum time from harvest to refrigeration, rate of refrigeration, and allowed time for refrigerated storage (NSSP, 2005).

In terms of prevention, effective vaccines would help in reducing the impact of *V. vulnificus* infection while certain antibiotics are important for protective immunity (LaFrentz and Shoemaker, 2015). In a recent study, American eels (*Anguilla rostrata*) were immunized with a recombinant bivalent expressed outer membrane protein (OMP) of *V. vulnificus* and *Aeromonas hydrophila* and the immunogenicity of this novel vaccine antigen was evaluated (Guo et al., 2015). Results showed that the expressed bivalent protein of OmpU of *V. vulnificus* and Omp porin II of *A. hydrophila* gave a good protective response against the two mentioned pathogens in the American eel under laboratory conditions (Guo et al., 2015). It is said that in order to develop an effective vaccine, priority should be given to immunogenic OMPs (Guo et al., 2015).

A passive immunization experiment was carried out recently to identify the role of antibodies against *V. vulnificus* infection (LaFrentz and Shoemaker, 2015). Results showed that specific antibodies have a role in protection of tilapia against infection and suggested that shared immunogenic antigens also play a role in protection against heterologous isolates (LaFrentz and Shoemaker, 2015). Another study targeted the C-terminal region (amino acid 3491–4701) of the pathogenic RTX (MARTX_Vv_ or RtxA1) protein and found that it successfully induced a protective immune response against *V. vulnificus* (Lee T. H. et al., 2014). Further, experiments with laboratory rodents injected with recombinant RtxA1-C protein with adjuvant also showed a long-lasting antibody response and significantly reduced bacterial load in the blood (Lee T. H. et al., 2014). Therefore, this study suggested that immunization against C-terminal region of RtxA1 may be an effective approach in the both prevention and therapy of *V. vulnificus* (Lee T. H. et al., 2014).

Given that the increased use of antibiotics in aquaculture resulted in the emergence of antibiotic resistance, considerable effort has been made in seeking alternative ways to control infections. Quorum sensing (QS) has been proposed to be one of the effective ways to attenuate virulence of organisms (Defoirdt et al., 2004). Quorum sensing is a process of bacterial cell-to-cell communication involving production, release, and detection of small signal molecules, which are also known as auto-inducers (Nealson et al., 1970; Ng and Bassler, 2009). These signal molecules are constantly produced and accumulated as the cells grow (Henke and Bassler, 2004; Antunes et al., 2011). Quorum sensing is also useful in monitoring bacterial cell densities and alter bacterial gene expression in a cell density-dependent manner (Ng and Bassler, 2009). Several techniques have been proposed in aiming to not only inactivating the signal molecule, but also to disrupt the signal detection in *Vibrios*. Defoirdt and colleagues has proposed a potential solution by distruption of QS in order to control the widespread antimicrobial resistance phenomenon (Defoirdt et al., 2004, 2011). The methods include (1) Inhibiting signal molecule biosynthesis, (2) Quorum sensing antagonist using both natural and synthetic halogenated furanones, antagonistic quorum sensing molecules, and non-specific exudates of higher plants and algae, (3) Chemical inhibition by oxidized halogen antimicrobials, (4) Disruption of signal molecule by bacterial lactonases and acylases, and (5) By applying quorum sensing agonists (Defoirdt et al., 2004).

QS disrupting compounds is also known as antipathogenic compounds because of its characteristics in attenuating virulence without affecting the growth of the bacteria. The QS disrupting compounds mentioned above were shown to have no effect toward the growth of the *Vibrios* and thus do not pose any selective pressure for the development of resistance. The fact that the probability of resistance development is smaller compare to the usage of antibiotics makes QS an attractive biocontrol strategy (Defoirdt et al., 2007).

## Conclusion

*V. vulnificus* is a potentially fatal infection that represents a great health care burden in certain countries. The emergence of multidrug resistant *V. vulnificus* with highly variable regional resistance profiles is a significant healthcare concern. It is essential to gain a more thorough understanding of the antibiotic resistance profile in different countries which is key to drawing up appropriate clinical practice guidelines for treatment and prevention of this potentially lethal pathogen. Efforts to create an effective vaccine are currently underway but are still in early stages, and identifying effective medical therapy seems to be the more immediate goal. Besides the effort of the researchers, both patients and clinicians also have to be educated in terms of effective prevention measures and antimicrobial resistance profile for an effective therapy in order to lower the fatality rate more convincingly. Ultimately, it is also key to find ways to limit antibiotic use in order to prevent increasing emergence of drug resistant microorganisms.

## Author contributions

SPH and VL performed the literature search and writing of the manuscript. CYD, NSAM, TMK, LHC, KGC, BHG, PP, and LHL provided critical review and insight to improve the writing. The research project was conceptualize by LHL.

### Conflict of interest statement

The authors declare that the research was conducted in the absence of any commercial or financial relationships that could be construed as a potential conflict of interest.
